# Structural Insights into the Nuclear Import of Haliotid Herpesvirus 1 Large Tegument Protein Homologue

**DOI:** 10.3390/v17091279

**Published:** 2025-09-20

**Authors:** Babu Kanti Nath, Crystall M. D. Swarbrick, Renate H. M. Schwab, Daryl Ariawan, Ole Tietz, Jade K. Forwood, Subir Sarker

**Affiliations:** 1 Biosecurity Research Program and Training Centre, Gulbali Institute, Charles Sturt University, Wagga Wagga, NSW 2678, Australia; cswarbrick@csu.edu.au (C.M.D.S.); rschwab@csu.edu.au (R.H.M.S.); 2 Dementia Research Centre, Macquarie Medical School, Faculty of Medicine, Health and Human Sciences, Macquarie University, North Ryde, Sydney, NSW 2109, Australia; daryl.ariawan@mq.edu.au (D.A.); ole.tietz@mq.edu.au (O.T.); 3 Training Hub Promoting Regional Industry and Innovation in Virology and Epidemiology, Gulbali Institute, Charles Sturt University, Wagga Wagga, NSW 2678, Australia; 4 Biomedical Sciences & Molecular Biology, College of Medicine and Dentistry, James Cook University, Townsville, QLD 4811, Australia; 5 Department of Microbiology, Anatomy, Physiology and Pharmacology, School of Agriculture, Biomedicine and Environment, La Trobe University, Melbourne, VIC 3086, Australia

**Keywords:** haliotid herpesvirus 1, nuclear trafficking, importins, crystallography

## Abstract

Abalone are highly susceptible to haliotid herpesvirus 1 (HaHV1), the causative agent of abalone viral ganglioneuritis (AVG), a re-emerging disease responsible for significant mortality events in both wild and farmed populations. Currently, there are no effective antiviral treatments or preventive measures available against HaHV1, which is partly due to the limited understanding of the immune responses and viral pathogenesis in this non-model marine invertebrate. This highlights the urgent need for novel intervention strategies, including investigations into the molecular mechanisms underlying HaHV1 infection. In other herpesviruses, the large tegument protein UL36 plays a crucial role in transporting the viral capsid to the host cell’s nuclear pore complex (NPC), mediated by N-terminal nuclear localization signals (NLSs). However, the nuclear import mechanism of UL36 homologue (UL36h) in HaHV1 remains largely uncharacterized. In this study, we identified and functionally characterized the NLS motif within HaHV1 UL36h and elucidated its interactions with the importin alpha (IMPα) nuclear import receptor. Through a combination of high-resolution crystallography and quantitative binding assays, we determined the key residues responsible for binding to IMPα and demonstrated isoform-specific variations in binding affinity. Our biochemical and structural analyses confirmed key interactions within the NLS that are essential for IMPα interactions. These findings advance our molecular understanding of HaHV1 host interactions and pave the way for the development of targeted antiviral strategies against abalone herpesvirus infection.

## 1. Introduction

Abalone herpesvirus 1 (AbHV1, also known as haliotid herpesvirus 1, HaHV1) is the etiological agent responsible for abalone viral ganglioneuritis (AVG), a highly contagious and lethal disease affecting abalone populations in Australia [1,2] and potentially other regions worldwide [3,4]. Outbreaks of AVG have resulted in severe mortality events among both farmed and wild abalone, with confirmed cases reported in the Australian states of Victoria and Tasmania [1,2,5]. The risk of AVG to aquaculture is worrisome, particularly in regard to the rapid expansion of global abalone farming. Between 2007 and 2017, worldwide aquaculture production increased more than fourfold, from approximately 41,000 metric tons to nearly 175,000 metric tons annually [6]. In contrast, wild-capture abalone harvests have remained comparatively modest, reaching about 6300 metric tons in 2017. Nevertheless, wild fisheries continue to play an important economic role in several regions, notably in Australia, where their estimated value was AU$152 million during 2023–2024 [7]. In response to the threat posed by this disease, Australia established routine surveillance programmes from 2011 onward, particularly prior to the interstate translocation and international trade of abalone stocks. Notably, in May 2021, a recreational diver discovered a cluster of dead abalone near Cape Nelson, Victoria, and subsequent diagnostic investigations confirmed an AVG outbreak (www.csiro.au). The virus was subsequently detected in the Portland region in mid-2021 and, by 2024, its presence was identified near Port MacDonnell in South Australia, leading to the enforcement of fishing restrictions in the Southern Zone (www.pir.sa.gov.au). Although several genetic variants of AbHV1 have been detected in Australia, their potential differences in pathogenicity and virulence profiles remain uncharacterized [2,5]. Phylogenetic analyses have demonstrated a high degree of sequence homology between AbHV1 and ostreid herpesvirus 1 (OsHV1), a related herpesvirus responsible for disease outbreaks in bivalves [8]. Based on these molecular findings, AbHV1 was subsequently reclassified as haliotid herpesvirus 1 (HaHV1) and designated as the type species of the newly established genus *Aurivirus*, within the family *Malacoherpesviridae* and the order *Herpesvirales* [9].

HaHV1 (species, *Aurivirus haliotidmalaco 1*), classified under the genus *Aurivirus*, is an enveloped double-stranded DNA virus with an icosahedral capsid approximately 100 nm in diameter [4]. Infection with HaHV1 results in acute mortality in abalone, associated with necrotizing ganglioneuritis, a neurological condition clinically recognized as AVG [1,2]. In *Haliotis diversicolor supertexta*, AVG manifests as extensive necrosis in the cerebral ganglia and peripheral nerves, particularly in the foot muscle and visceral tissues, typically accompanied by hemocyte infiltration and pronounced inflammatory responses. The continuing threat of AVG to abalone aquaculture is of major concern, especially given the absence of effective antiviral therapies. Current management approaches rely primarily on vaccination, which provides limited protection and does not eliminate infection risk. In light of these challenges, a deeper understanding of the molecular processes governing HaHV1 infection, particularly its nuclear trafficking pathways, is essential. Such knowledge could facilitate the development of targeted antiviral strategies and enhance disease control efforts in affected aquaculture industries.

The importin α/β1 (IMPα/β1) pathway is one of the most extensively studied mechanisms governing the nuclear import of proteins. In this process, one of the seven known isoforms of importin α (IMPα) recognizes and binds to cargo proteins containing a classical nuclear localization signal (cNLS). This cargo–IMPα complex subsequently associates with importin β1 (IMPβ1) via the N-terminal importin β–binding (IBB) domain of IMPα, forming a trimeric complex [10,11]. This assembly is then transported through the nuclear pore complex (NPC), with IMPβ1 interacting with phenylalanine-glycine (FG) repeat motifs present in nucleoporins lining the NPC channel. Once inside the nucleus, RanGTP binds to IMPβ1, triggering conformational changes that lead to the disassembly of the import complex and release of the cargo protein. Following cargo release, both IMPα and IMPβ1 are recycled back to the cytoplasm for subsequent rounds of nuclear import [12,13,14]. IMPα isoforms are classified into three subfamilies based on sequence similarity and phylogenetic relationships. In humans, the α1 subfamily includes IMPα5 (KPNA1), IMPα6 (KPNA5), and IMPα7 (KPNA6); the α2 subfamily comprises IMPα1 (KPNA2) and IMPα8 (KPNA7); while the α3 subfamily consists of IMPα3 (KPNA4) and IMPα4 (KPNA3) [15,16]). Structurally, the central region of IMPα contains 10 tandem Armadillo (Arm) repeat motifs, each approximately 42–43 amino acids in length and predominantly composed of hydrophobic residues. These Arm repeats form a binding groove that accommodates cNLS-containing cargoes. Two primary binding pockets within the Arm repeats, known as the major and minor sites, mediate this interaction. The major site, spanning Arm repeats 2 to 4, accommodates residues designated P1 to P5 of the cNLS, while the minor site, located within Arm repeats 6 to 8, binds positions P1′ to P4′. Typically, monopartite cNLSs, such as that of the SV40 large T antigen, engage only the major site, whereas bipartite cNLSs, like those in nucleoplasmin, simultaneously interact with both binding sites [17].

For successful infection, all herpesvirus capsids must traverse the cytoplasm and dock at the NPCs, where capsid remodelling events, through mechanisms that remain incompletely understood, enable the release and delivery of the viral genome into the nucleus [18]. This nuclear delivery process is most extensively characterized in herpes simplex virus type 1 (HSV-1), where it initiates the transcription of immediate-early viral genes essential for establishing infection [19]. A pivotal component in this process is VP1-2, a large tegument protein encoded by the UL36 gene, which is both highly conserved and functionally indispensable across herpesvirus species [20,21,22,23]. VP1-2 is a multifunctional protein involved in several critical stages of the viral life cycle, including capsid trafficking, genome delivery, and virion assembly [20,24,25]. Previous studies have identified a functional NLS within the N-terminal region of VP1-2, positioned adjacent to its ubiquitin-specific protease (USP) domain in HSV-1 (amino acid residues 400–420: GLPKRRRPTWTPPSSVEDLTS) [25,26,27]. Deletion of this NLS was shown to disrupt capsid docking at the NPCs and inhibit the onset of viral gene expression, while having no effect on the assembly or egress of extracellular virions [25]. These findings suggest that the nuclear targeting function of VP1-2 is essential, specifically during the early stages of herpesvirus infection. Furthermore, the positional conservation of this NLS among several herpesvirus orthologues [26] implies a conserved and indispensable role for VP1-2 nuclear trafficking in the early infection dynamics across the herpesvirus family [27].

To date, no study has characterized the specific importin proteins (IMPs) responsible for recognizing and binding the NLSs within large tegument proteins of α-Herpesviridae members, nor has the functional significance of these NLS motifs been investigated in viruses infecting abalone. In this study, we aimed to characterize the structural and functional properties of the predicted NLS within the UL36h of HaHV1 and to elucidate the mechanisms governing its nuclear import. To achieve this, we employed an integrated approach combining structural biology techniques with quantitative biochemical assays to explore the molecular interactions involved in HaHV1 UL36h nuclear trafficking.

## 2. Materials and Methods

### 2.1. Retrieval and Analysis of HaHV1 Genomic Sequences

Complete genome sequences of HaHV1/AbHV1, were obtained from the GenBank database (GenBank accession nos. JX453331 and MW412419). A set of other selected herpesviruses were used to compare with HaHV1 and analyze using Geneious Prime (version 2023.1.1). The majority of coding regions within the HaHV1 genome were annotated as hypothetical proteins. One open reading frame (designated ORF18 in accession JX453331 and ORF15 in MW412419) was tentatively identified as a homologue of the large tegument protein UL36. To compare homologous sequences, multiple sequence alignment of the predicted UL36 protein homologue (UL36h) was performed using MAFFT (version 7.450), applying the G-INS-i strategy with a gap opening penalty of 1.53 and an offset value of 0.123.

### 2.2. Peptide and Gene Construct Design and Synthesis

The UL36h protein sequence from accession JX453331 was used to predict potential nuclear localization signals (NLS) using the cNLS Mapper algorithm [28]. This analysis identified a monopartite NLS between residues 1491 and 1500, with the sequence 1491-ETKKRRRILE-1500 and a prediction score of 8.0, indicating strong nuclear import potential. Based on this prediction, peptide and gene constructs containing the NLS were designed. Synthetic peptides corresponding to the predicted NLS and its mutants, each modified with an FITC/Ahx group at the N-terminus, were synthesized at Macquarie University, Sydney, Australia, using standard Fmoc-based solid-phase peptide synthesis protocols on a CEM Liberty Blue™ automated synthesizer (CEM, Matthews, NC, USA) according to previously published procedure [29]. Briefly, Rink amide resin was pre-swelled in a 1:1 mixture of dimethylformamide (DMF) and dichloromethane (DCM) for 1 h prior to synthesis. Amino acids were dissolved in DMF at a final concentration of 0.2 M and coupled sequentially from the C- to N-terminus at 90 °C for 3 min using five equivalents of amino acid, ten equivalents of *N*,*N*′-Diisopropylcarbodiimide (DIC) in DMF as activator, and five equivalents of Oxyma/DIPEA (0.5 M/0.05 M in DMF) as base. Fmoc deprotection was performed with 20% piperidine in DMF at 90 °C for 2 min, followed by resin washing in DMF. Double couplings were employed for arginine residues to ensure complete incorporation. After final Fmoc removal of the N-terminal aminohexanoic acid (Ahx), the resin was washed and subjected to overnight coupling with 3 equivalents of fluorescein isothiocyanate (FITC) and 6 equivalents of DIPEA in DMF. Following FITC coupling, the peptides were sequentially washed with DMF, DCM, and methanol before cleavage from the resin using a cocktail containing 92.5% trifluoroacetic acid (TFA), 2.5% triisopropylsilane (TIPS), 2.5% thioanisole, and 2.5% water for 3–6 h at room temperature. Peptides were then precipitated in ice-cold diethyl ether, dissolved in water, freeze-dried, and purified using a Shimadzu LC-20AD high-performance liquid chromatography (HPLC) system (Shimadzu, Nakagyo-ku, Kyoto, Japan). Peptide identity and purity were confirmed by mass spectrometry on a Shimadzu LCMS-8050 instrument operating in positive electrospray ionization mode with a Polaris 3 C18-A 150 × 4.6 mm column (Agilent Technologies, Santa Clara, CA, USA).

Mutant derivatives of the FITC-labelled NLS peptide were designed based on the structural interface of IMPα2 with classical NLS sequences. Recombinant N-terminally truncated isoforms of importin α1 (hIMPα1ΔIBB), α2 (mIMPα1ΔIBB), and α3 (hIMPα3ΔIBB)-lacking the autoinhibitory importin-β binding (IBB) domain-each incorporating a His-tag and TEV protease cleavage site, along with importin β1 encoded in the pMCSG21 vector, were produced as previously described [30,31].

### 2.3. Recombinant Expression and Purification of Importin Isoforms

Recombinant overexpression of human IMPα1ΔIBB (UniProt: P52292), mouse IMPα2ΔIBB (UniProt: P52293), human IMPα3ΔIBB (UniProt: O00629), and mouse IMPβ1 (Uniprot: P70168) was performed in *Escherichia coli* pLysS cells using an auto-induction method [32]. Cultures were incubated at room temperature for 36 h, after which bacterial cells were harvested by centrifugation at 5232× *g* for 30 min. Cell pellets were resuspended in His buffer A (50 mM phosphate buffer, 300 mM NaCl, 20 mM imidazole, pH 8.0) at a ratio of 20 mL per 2 L of culture and subjected to three freeze–thaw cycles to facilitate initial cell lysis. Further lysis was achieved by adding 2 mL of lysozyme solution (20 mg/mL, Sigma-Aldrich, St. Louis, MI, USA) and 20 µL of DNase (50 mg/mL, Sigma-Aldrich, USA) per 35 mL of cell suspension, followed by incubation on a tube roller at room temperature for 1 h. The lysates were clarified by centrifugation at 11,269× *g* for 45 min, and the resulting supernatants were filtered through a 0.45 µm low protein-binding membrane filter to obtain soluble protein extracts. The filtered lysates were applied to a 5 mL HisTrap HP affinity chromatography column (GE Healthcare, Chicago, IL, USA) pre-equilibrated with His buffer A, using an AKTA purifier FPLC system (GE Healthcare, Chicago, IL, USA). Bound proteins were washed with twenty column volumes of His buffer A, and subsequently eluted with a linear imidazole gradient (20 mM to 500 mM, ChemSupply, Gillman, SA, Australia). Eluted fractions containing the target proteins were pooled for further purification. Size-exclusion chromatography was performed using a HiLoad 26/60 Superdex 200 column (GE Healthcare, Chicago, IL, USA) pre-equilibrated with GST buffer A (50 mM Tris, 125 mM NaCl, pH 8.0). Fractions corresponding to the expected molecular weights of the recombinant importins were collected, concentrated using an Amicon Ultra centrifugal filter with a 10 kDa molecular weight cut-off (Merck Millipore, Burlington, MA, USA), aliquoted, and stored at −80 °C until use. Protein purity and integrity were evaluated by SDS-PAGE, with samples run at 165 V for 35 min on a 4–12% Bis-Tris Plus gel (Thermo Fisher Scientific, Waltham, MA, USA). Gels were stained with Coomassie Brilliant Blue to visualize protein bands prior to downstream applications.

### 2.4. Crystallization, Data Collection and Structure Determination

Crystallization of IMPα2 was carried out using the hanging drop vapour diffusion method at 23 °C, following the protocol described previously [33]. Briefly, equal volumes of protein solution and reservoir solution (0.6 M sodium citrate, 0.1 M HEPES pH 7.0, and 10 mM DTT) were mixed and equilibrated against 300 μL of reservoir solution. Rod-shaped crystals typically appeared within 48 h of incubation. Crystals were soaked with the desired peptide and cryoprotected in the reservoir solution containing 20% glycerol, before being flash frozen in liquid nitrogen. X-ray diffraction data were obtained from the Australian Synchrotron on the MX2 macromolecular beam lines [34] using the Eiger 16 M detector. Data were indexed and integrated using XDS [35]. Merging, space group assignment, scaling and Rfree calculations were performed using AIMLESS within CCP4 [36]. Final model building and refinement were performed using iterative cycles of COOT [37] and Phenix [38]. Phasing was performed using molecular replacement in Phaser [39] and PDB code 6BW1 was used as the search model for IMPα2. The finalized model was validated and deposited with the PDB (9PYR).

### 2.5. Fluorescence Polarization Assay

Synthetic FITC-labelled peptide (2 nM final concentration) was incubated with two-fold serial dilutions of importin isoforms, starting at 20 μM, across 23 wells in a final volume of 200 μL per well in GST buffer A (50 mM Tris, 125 mM NaCl, pH 8.0). Fluorescence polarization measurements were performed using a CLARIOstar Plus plate reader (BMG Labtech, Allmendgrün, Ortenberg, Germany) in 96-well black Fluotrac microplates (Greiner Bio-One, Kremsmünster, Austria). Each assay was conducted in triplicate, each containing a negative control (no importin). Data was analyzed by non-linear regression using GraphPad Prism (Prism 9, Version 9.3.1).

### 2.6. Electro-Mobility Shift Assay (EMSA)

The FITC-labelled peptide (10 μM) was incubated with 20 μM of each importin α isoform in a total reaction volume of 20 μL. The reaction mixture was supplemented with 3 μL of 50% (*v*/*v*) glycerol, with the remaining volume adjusted using GST buffer A (50 mM Tris, 125 mM NaCl, pH 8.0). Samples were electrophoresed on a 1% (*w*/*v*) agarose gel prepared in TB buffer (45 mM Trizma base, 45 mM boric acid, pH ~8.5) at 80 V for 2 h. Fluorescent images of the gels were captured using a SYBR Green filter on a Bio-Rad Gel Doc imaging system (Bio-Rad Laboratories, Hercules, CA, USA) to visualize the FITC-tagged peptide and its complexes. Following fluorescence imaging, the gel was stained with Coomassie Brilliant Blue R-250 staining solution (40% ethanol, 10% glacial acetic acid, 0.2% Coomassie Brilliant Blue) for 5 min at room temperature. Destaining was performed overnight in a solution of 10% ethanol and 10% glacial acetic acid. Gels were imaged again using the same imaging system to assess total protein migration and complex formation.

## 3. Results

### 3.1. Genetic Variability of the HaHV1 UL36 Homologue Gene

The amino acid sequences of the full-length UL36 homologue (UL36h) from HaHV1 (ORF18 in GenBank accession JX453331 and ORF15 in MW412419) exhibited a high degree of similarity, with 99.17% sequence identity. Notably, the predicted nuclear localization signal (NLS) region was completely conserved between the two sequences (100% identity). Although HaHV1 has been classified under the recently proposed genus *Aurivirus*, the UL36h protein, comprising approximately 1555 amino acids-shares less than 10% sequence identity with the UL36 proteins of other known herpesviruses, indicating substantial evolutionary divergence.

### 3.2. Biochemical Determination of HaHV1 NLS Preference for Importin α Isoforms

**Figure 1:**
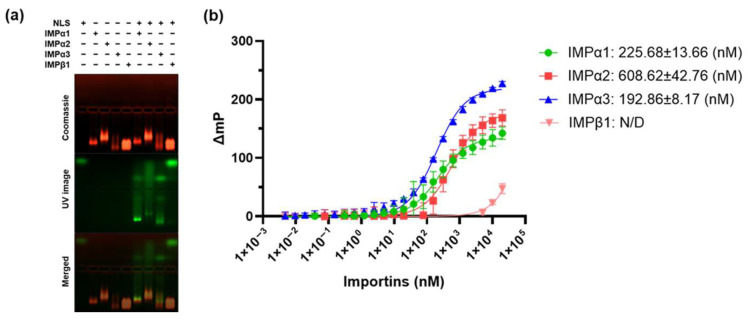
HaHV1 UL36h NLS binds to several importins (IMPs) (**a**) EMSA showing binding of HaHV1 UL36h NLS to IMPα isoforms. NLS peptides contain a FITC and Ahx linker and were visualized by excitation with an UV lamp (green). Proteins were stained using Coomassie blue stain (red). EMSA results are representative of three independent experiments. (**b**) FP assay measuring the binding affinity between the HaHV1 UL36h NLS and respective IMPs isoforms. Data shown are mean ± standard error of the mean (SE) relative to three independent experiments. Data were used to calculate the Kd, as described in Section 2.

To investigate whether the predicted NLS within the HaHV1 UL36h protein could interact with host nuclear import receptors, a series of biochemical binding assays were conducted. Electrophoretic mobility shift assays (EMSA) were initially performed to qualitatively examine the binding interactions between the synthetic HaHV1 NLS peptide and various importin isoforms, including members of the importin α (IMPα) family (α1, α2, and α3), lacking the importin β-binding domain (ΔIBB), and importin β1 (IMPβ1). Results from the EMSA’s consistently demonstrated that the HaHV1 NLS formed detectable complexes with all tested IMPα isoforms, whereas no interaction was observed with IMPβ1 (Figure 1a).

To quantitatively characterize these interactions and determine binding affinities, fluorescence polarization (FP) assays were subsequently performed following established protocols [40,41,42,43,44,45] (Figure 1b). The monopartite HaHV1 NLS exhibited measurable affinities for each IMPα isoform tested (Figure 1b). Among these, the strongest interaction was observed with IMPα3 (Kd = 192.86 nM), followed by IMPα1 (Kd = 225.68 nM), and IMPα2 (Kd = 608.62 nM). Collectively, these findings confirm that the HaHV1 UL36h NLS specifically interacts with IMPα isoforms with differing affinities and suggest that nuclear import of the UL36h protein is mediated through the classical IMPα-dependent pathway.

### 3.3. The High-Resolution Crystal Structure Reveals the Binding Interface of IMPα and HaHV1 NLS

To explore the molecular mechanisms by which HaHV1 enters the nucleus, we crystallized the NLS of UL36h (residues 1491–1500) in complex with the nuclear import protein mouse IMPα2. Protein crystallization was performed using the hanging-drop method and large rod-shaped crystals were obtained in the presence of the NLS. Crystals were diffracted at the Australian synchrotron on the MX2 beamline and data collected to 2.6 Å. Data were indexed in the space group P2_1_ 2_1_ 2_1_ with the unit cell parameters 78.5 90.8 100.9. The structure was solved in Phaser [39] by molecular replacement with PDB 6BW1 as the search model (detailed statistics can be seen under Table 1).

**Figure 2:**
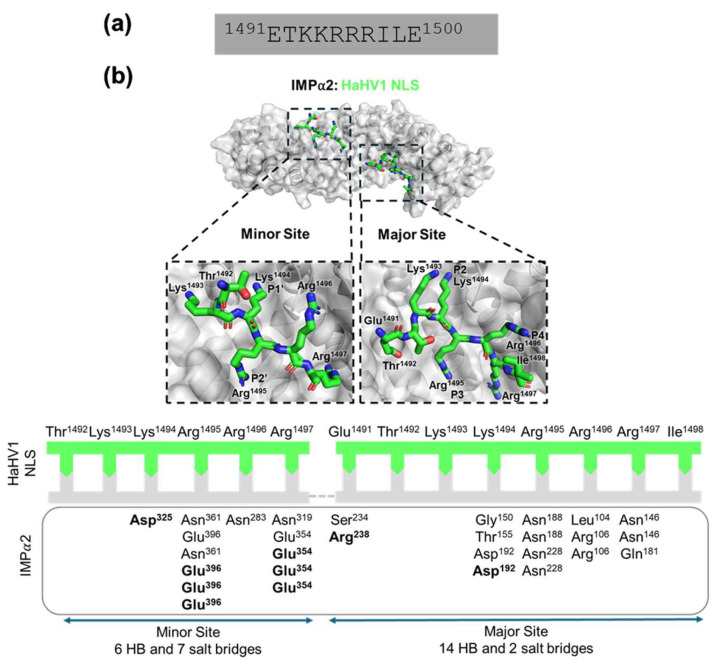
Crystal structure and binding interactions of HaHV1 NLS in complex with mouse IMPα2. (**a**) Sequence of the predicted NLS of HaHV1. (**b**) Top panel: Schematic overview of the HaHV1 protein and structure of HaHV1 NLS (green sticks) and IMPα2 (grey surface) complex resolved to 2.6 Å resolution. The zoomed-in images illustrate critical residues of HaHV1 NLS binding in both minor and major IMPα2 sites. This structure has been deposited in the PDB and given the code: 9PYR. Bottom panel: Simplified representation of IMPα2 and HaHV1 NLS binding interactions. The HaHV1 NLS (green line) residues bound to IMPα2 (grey box) are indicated through complementary arrows. Residues in bold denote salt bridge and non-bold residues indicate hydrogen bonds identified using the PDBePISA server.

The IMPα:NLS structure contained one molecule of IMPα with two chains of HaHV1 NLS bound. Iterative rounds of refinement and modelling were performed with Phenix [38] and COOT [37,38], with a final R_work_/R_free_ of 0.18/0.21. The full data collection and refinement statistics are listed in Table 1. The final IMPα:NLS model contained IMPα (residues 72–497), with HaHV1 NLS (residues ^1491^ETKKRRRI^1498^) bound in both the major (ARM repeats 2–4) and minor (ARM repeats 7–8) sites of IMPα. Structural characterization of the interface revealed that HaHV1 UL36h binds to the IMPα major site with a canonical monopartite NLS, and a well-characterized lysine at the P2 site (Figure 2). The protein complex was analyzed using PDBePISA and the interface at the minor site comprises an interface of 608.2 Å ^2^ mediated by six hydrogen bonds and seven salt bridges, whilst the major site comprises an interface of 779.4 Å ^2^ and is mediated by fourteen hydrogen bonds and two salt bridges (Table 2).

Within the IMP⍺ major site, HaHV1-NLS Glu^1491^ interacts with IMP⍺2 Ser^234^ and forms a salt bridge with Arg^238^ (Figure 2b; Table 2). HaHV1-NLS Lys^1494^ binds IMP⍺2 residues Asp^192^, Gly^150^ and Thr^155^, forming a salt bridge with Asp^192^. HaHV1-NLS Arg^1495^ forms two hydrogen bonds with each IMP⍺ Asn^188^ and Asn^228^, and HaHV1-NLS Arg^1496^ forms two hydrogen bonds with Arg^106^ and one hydrogen bond with Leu^104^. Finally, HaHV1-NLS Arg^1497^ binds IMP⍺2 by forming hydrogen bonds with Asn^146^ and Gln^181^. We also observed a secondary binding of the HaHV1-NLS at the minor site of IMPα (Figure 2b; Table 2); however, this is likely an artefact due to the high concentration of peptide required for crystallization, and similar to what has been observed and described previously for other monopartite NLSs, such as the SV40 LTA NLS bound to mouse and yeast IMPαs [46,47] and other NLSs [48]. Moreover, the average B-factors were 69.8 and 89.8 Å^2^ for the major and minor sites, respectively, whilst the average B-factor for IMPα (residues 70–529) is 65.6 Å^2^.

### 3.4. Mutational Studies Confirm Monopartite Nature of HaHV1 NLS

**Figure 3:**
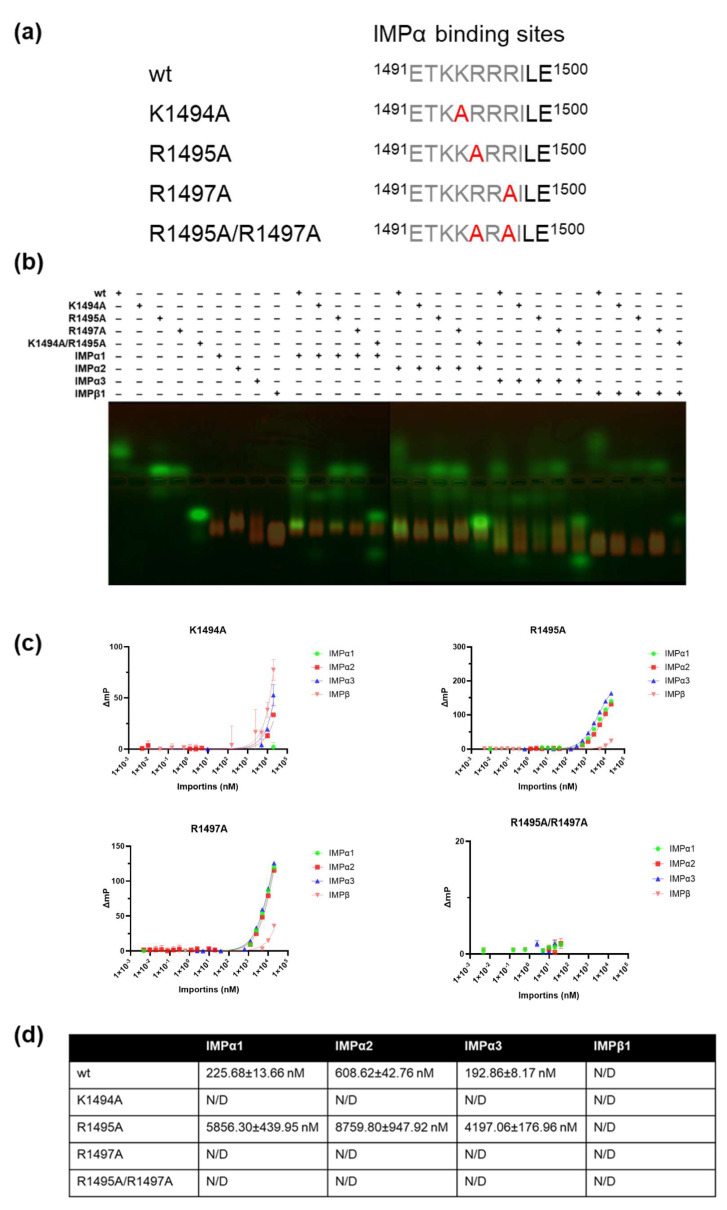
Binding affinities of IMP isoforms with HaHV1 UL36h NLS mutants. (**a**) Schematic representation of HaHV1 tegument protein NLS mutants. (**b**) EMSA demonstrates that each of the NLS mutant peptides possesses very poor binding affinity to IMPα isoforms. All peptides contain an N-terminal FITC label and Ahx linker. Proteins were stained using Coomassie blue stain (artificially coloured red for more clarity), and the overlay image FITC tag peptide and Coomassie stained IMPαs is represented. EMSA results are representative of three independent experiments. (**c**) FP assay measuring the direct binding between the NLS mutants and the indicated IMPs isoforms. (**d**) Kd values shown are mean ± standard error of the mean (SEM) relative to three independent experiments. Non-linear regression was used to calculate the Kd in Graphpad Prism, as described in Section 2.

Since the HaHV1 sequence, ^1491^ETKKRRRILE^1500^, consists of a single stretch of basic amino acids observed interacting with IMPα2 (Figure 3a), we hypothesized that this region may function as a monopartite NLS. To test this, we investigated the contribution of individual interacting residues to the binding of the HaHV1 NLS to multiple IMPα isoforms (Figure 3a) using both EMSA (Figure 3b) and FP (Figure 3c) assays. Substitutions at positions K^1494^A, R^1495^A, and R^1497^A, which correspond to key contacts within the P2, P3, and P5 pockets of the IMPα major binding site, markedly reduced the co-migration of the mutant peptides with all tested IMPα isoforms in EMSA (Figure 3b). Consistently, these substitutions also caused a significant increase in the dissociation constant (K_D_) in FP assays (Figure 3c,d), indicating diminished binding affinity. Because structural analysis revealed that residue R^1495^ interacts with the P2′ residue (Glu^396^) of the IMPα2 minor site through three salt bridges, while residue R^1497^ forms strong interactions with the minor site residue Glu^354^, also via three salt bridges. Preliminary single site mutations at either R^1495^ or R^1497^ partially reduced binding affinity with importins but did not completely abolish the interaction. To further validate the binding interface, we generated a double mutant (R^1495^A/R^1497^A). The R^1495^A/R^1497^A double mutant displayed a markedly reduced binding affinity compared to both the wild-type peptide and the single site mutants, indicating a synergistic effect of these residues in mediating importin recognition. These results confirm that R^1495^ and R^1497^ are also critical determinants of UL36h nuclear import activity. Collectively, these findings confirm the critical role of residues 1494–1497 in mediating high-affinity interactions at the major binding site of IMPα and suggest that this interaction is essential for the effective recognition of the HaHV1 NLS by IMPα isoforms.

## 4. Discussion

Herpesviruses are nuclear-replicating DNA viruses that rely on precise regulation of viral protein localization and function within the host cell to establish productive infection, making the characterization of these processes crucial for developing effective antiviral therapies [49,50]. Studies in HSV-1 have revealed a highly organized pattern of subcellular distribution among viral proteins, with approximately 21 proteins localized to the cytoplasm or associated membranes, 16 confined to the nucleus or specific subnuclear compartments, and several others distributed across both compartments [51]. Notably, most herpesvirus envelope proteins are retained in the cytoplasm, whereas capsid-associated proteins predominantly accumulate within the nucleus. This compartmentalization reflects the functional requirements of each protein during different stages of the viral life cycle [51]. Among these, VP1-2, a large and highly conserved tegument protein encoded by the UL36 gene, plays a pivotal role in several key steps of herpesvirus replication, particularly in the nuclear delivery of viral genomes following entry [25]. Previous studies have established that while the NLS within VP1-2 is dispensable for virion assembly and egress, it is indispensable for the efficient docking of incoming capsids at the NPC, a critical prerequisite for initiating viral gene expression and replication. The positional conservation of this NLS motif across diverse herpesvirus species suggests that this nuclear targeting mechanism is likely a shared and essential feature within the *Herpesviridae* family [25,27].

HSV-1 is known to employ multiple nuclear import strategies to ensure the efficient localization of viral proteins to the host cell nucleus during infection [52,53,54]. For instance, HSV-1 utilizes the classical importin α/β1 (IMPα/β1) pathway to mediate the nuclear import of its DNA polymerase processivity factor UL42 [55], while other proteins, such as the tegument protein VP16, achieve nuclear localization through interactions with host cell factor HCF-1 [56]. Despite advances in understanding herpesvirus nuclear trafficking, the nuclear import mechanisms of HaHV-1 have remained largely uncharacterized. To address this gap, we investigated the classical NLS within the UL36h of HaHV1, applying a combination of structural, biophysical, and biochemical approaches to elucidate its interactions with host importins (IMPs). Through structural characterization of the UL36h-IMPα2 complex, we identified a unique monopartite NLS motif within the HaHV1 UL36h. Subsequent biochemical assays using a fluorescein isothiocyanate (FITC)-labelled NLS peptide confirmed binding interactions with multiple tested IMPα isoforms. Notably, the NLS displayed the highest affinity for IMPα3, followed by IMPα1 and IMPα2. In the canonical classical import pathway, proteins bearing a classical NLS are recognized by IMPα, which subsequently forms a heterodimer with IMPβ1 to mediate nuclear import through the NPC. In contrast, non-classical pathways bypass this machinery, relying on mechanisms such as passive diffusion, direct interactions with nucleoporins, or IMPβ1-dependent/IMPα-independent import routes [57,58]. To investigate whether the interaction between HaHV1 NLS and IMPα conformed to classical paradigms, we determined high-resolution crystal structures of the binding interface between IMPα2 and the HaHV1 NLS peptide.

The crystal structure of the IMPα2:NLS complex revealed one molecule of IMPα2 and two peptide chains corresponding to the HaHV1 NLS. The final refined model included residues 72–498 of IMPα2, with the HaHV1 NLS (residues 1491–1500: ETKKRRRILE) bound at the classical major binding site, located within ARM repeats 2–4 of IMPα2. Structural examination of the interaction interface demonstrated that the HaHV1 UL36h engages IMPα2 via a canonical monopartite NLS motif. Notably, the lysine residue at position 1494 (K^1494^) occupied the critical P2 binding pocket, a hallmark of classical NLS-IMPα interactions (Figure 2b). This binding mode mirrors that of well-characterized monopartite NLS sequences, such as the SV40 large T antigen [47], and is consistent with structural features observed in other viral and host protein complexes [59]. Among members of the *Alphaherpesvirinae* subfamily, including HSV, pseudorabies virus (PRV), and equine herpesvirus 1 (EHV1), the VP1-2 also features continuous N-terminal basic clusters that function as monopartite NLS motifs. These motifs typically include an extended linker region accompanied by a proline/serine/threonine (P/S/T)-rich segment, which is hypothesized to modulate NLS function under specific physiological or cellular conditions [27]. The linker region between upstream and downstream basic clusters has been shown to interact with IMPα and may influence the overall binding affinity or transport efficiency. While such structural organization may be non-essential for viral replication in cell culture systems, it is likely to play a crucial role in optimizing nuclear import, genome delivery, and replication efficiency within the complex cellular environments of an infected host, particularly in neurons and other highly polarized cell types [27].

**Figure 4:**
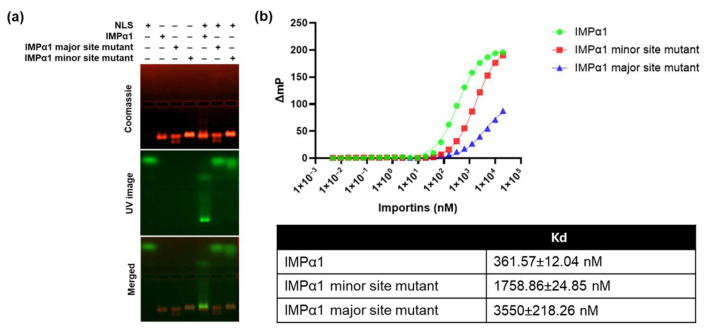
HaHV1 UL36h NLS does not bind to IMPα1 major site mutant (**a**) EMSA showing no binding affinity of HaHV1 large tegument protein NLS to IMPα1 major and minor site mutants. HaHV1 UL36h NLS peptides contain an N-terminal FITC label and Ahx linker and were visualized by excitation with an UV lamp (green). Proteins were stained using Coomassie blue stain (red). EMSA results are representative of three independent experiments. (**b**) FP assay measuring the binding affinity between the HaHV1 UL36h NLS and IMPα1 major and minor site mutants. Data shown are mean ± standard error of the mean (SEM) relative to three independent experiments. Non-linear regression was used to calculate K_D_ values in GraphPad Prism, as described in Section 2.

EMSA assessing mutations within the HaHV1 NLS region that interacts with the major binding site of IMPα revealed that these mutant variants displayed markedly reduced binding to all tested IMPα isoforms (Figure 3a,b). This observation indicates that these residues likely constitute a functional NLS within the cellular environment. Complementary FP analyses further demonstrated that individual substitutions of key basic residues—Lys^1494^, Arg^1495^, and Arg^1497^-to Ala significantly impaired binding to all IMPα isoforms examined (Figure 3c,d). Moreover, targeted mutation of a critical residue within IMPα1’s major binding site (Asp^192^ to Lys; D^192^K) considerably diminished NLS co-migration in EMSA (Figure 4a) and substantially decreased binding affinity, as reflected by higher dissociation constants (Kd) in FP assays (Figure 4b). A similar but less pronounced effect was observed following substitution of Glu^396^ to Arg (E^396^R) within the minor binding site of IMPα1, which also reduced co-migration and affinity relative to wild-type IMPα1 (Figure 4a,b). These data suggest that the HaHV1 NLS engages both the major and minor binding pockets of IMPα1, with a clear preference for the major site, as evidenced by the greater impact of the D^192^K mutation on binding affinity. Collectively, these findings reinforce the concept that HaHV1 UL36h NLS residues show preferential interaction with the major binding groove of IMPα isoforms. This is consistent with earlier observations in other herpesviruses, where detergent-extracted HSV-1 virions were shown to associate with NPCs in vitro, a process partially inhibited by antibodies targeting nucleoporins or IMPβ1 [52]. While specific viral receptors mediating this interaction remain unidentified, the binding specificity of viral NLS motifs for different IMP isoforms is still not fully determined. Given that structural analyses have shown the NLS-binding groove of IMPα to be highly conserved across isoforms [60,61,62,63], it is likely that subtle variations in NLS sequences and IMPα isoform expression patterns influence cargo selectivity and transport dynamics. Although the abalone genome is not yet fully characterized, our BLASTp analysis of human IMPα isoforms identified IMPα1-, IMPα4-, and IMPα6-like proteins in abalone. Sequence comparison revealed that human and abalone IMPα isoforms share 57–75% amino acid identity. Furthermore, analysis of the major and minor binding sites demonstrated a high degree of conservation between abalone and human IMPα (Supplementary Figure S1). Given that the IMP’s are relatively conserved and abalone IMPα proteins are not currently available in our laboratory, we relied on the human and mouse IMPα isoforms for structural and biochemical assays.

To investigate the conservation of NLS recognition between mouse and abalone importins, residues 68–520 of *Haliotis asinina* IMP⍺1 (NCBI accession number: XP_067674051.1) were modelled using AlphaFold 3. Structural comparison revealed a high degree of similarity between *H. asinina* IMP⍺1 and mouse IMP⍺2 (Supplementary Figure S2). Superimposition of the two structures showed that the overall ARM repeat fold was preserved, with both proteins adopting the canonical IMP⍺ architecture (Supplementary Figure S2a). The modelled structure of *H. asinina* IMP⍺1 aligned closely with the mouse IMP⍺2 crystal structure, particularly at the major NLS-binding site where the HaHV1 NLS peptide was docked. A detailed view of the NLS-binding interface demonstrated that the critical residues mediating NLS recognition in mouse IMP⍺2 were strictly conserved in *H. asinina* IMP⍺1 (Supplementary Figure S2b). These residues, located within the major binding pocket, established equivalent interactions with the NLS peptide, suggesting functional conservation of NLS recognition between mouse and abalone importins. First limitation of this study is that binding assays could not be performed with the full-length UL36h protein due to its large size, the complexity of fluorescent labelling and associated technical challenges in recombinant expression. Instead, we used a fluorescently labelled synthetic peptide corresponding to the predicted NLS, which specifically interacted with nuclear import receptors, providing functional evidence for its role in nuclear import. Future studies using truncated or full length constructs will be important to further define the mechanistic details of UL36h–importin interactions. Another limitation of this study is that we used human and mouse IMPα isoforms rather than abalone derived proteins. Although, IMPα proteins are highly conserved across metazoans, particularly in the ARM repeat domain that mediates NLS binding, and cross species compatibility of IMPα–NLS interactions has been widely demonstrated, it would be important to investigate further using abalone IMPα. Additionally, we have also successfully applied human/mouse IMPα in studies of viral NLSs from frog and psittacine adenoviruses [41,45], supporting the validity of this approach. Nonetheless, future work with abalone IMPα will be essential to confirm host specific interactions and strengthen the virological significance of our findings.

## 5. Conclusions

This study demonstrates that the predicted C-terminal NLS within the HaHV1 UL36h facilitates nuclear import via the IMPα/β1-dependent pathway, while also suggesting the potential involvement of alternative nuclear import mechanisms. The results indicate that herpesviruses may utilize multiple, possibly species-specific, nuclear transport routes to achieve efficient nuclear targeting. To further elucidate the mechanisms underlying this process, future studies should focus on detailed subcellular localization analyses and the exploration of additional nuclear import pathways. Moreover, although this investigation centred on the HaHV1 UL36h, it would be valuable to assess whether comparable nuclear import strategies are conserved across other alphaherpesviruses by examining their respective UL36. Collectively, these preliminary findings enhance our understanding of HaHV1 molecular biology and provide insights that may guide future investigation into antiviral strategies, while also contributing to the broader field of animal herpesviruses.

## Figures and Tables

**Table 1:** Data collection and refinement statistics for structure of importin-α2 in complex with HaHV1 NLS.

HaHV1 NLS1 and Mouse Importin-α2 (PDB Code: 9PYR)	
Data collection (high-resolution statistics in parentheses)	
Wavelength (Å)	0.95374
Data collection temperature (K)	298
Detector Type	Dectris EIGER × 16 M
Detector	Pixel
Resolution range (Å)	29.71–2.60
Space group	P2_1_ 2_1_ 2_1_
Unit cell (Å); (^o^)	78.5 90.8 100.9; 90 90 90
Total reflections	173,168
Unique reflections	22,759 (2745)
Multiplicity	5.3 (5.5)
Completeness (%)	99.7 (99.9)
Mean I/σ (I)	11.2 (2.6)
Wilson B-factor Å^2^	51.84
R_pim_	0.041 (0.328)
Refinement	
R_work_	0.18
R_free_	0.21
No. of non-hydrogen atoms	3355
Macromolecules	3
Solvent	14
Protein residues	440
Bond length r.m.s.d (Å)	0.006
Bond angle r.m.s.d (^o^)	0.848
Ramachandran favoured (%)	97.0
Ramachandran allowed (%)	3.0
Ramachandran outliers (%)	0.0

**Table 2:** Hydrogen bond and salt bridge interactions between HaHV1 NLS and mouse IMPα2.

HaHV1 NLS	Mouse IMPα2
**Hydrogen bonds (major site)**	
GLU^1491^ [OE1]	SER^234^ [OG]
LYS^1494^ [HZ2]	GLY^150^ [O]
LYS^1494^ [HZ3]	THR^155^ [OG1]
LYS^1494^ [HZ1]	ASP^192^ [OD1]
ARG^1495^ [O]	ASN^188^ [HD21]
ARG^1495^ [H]	ASN^188^ [OD1]
ARG^1495^ [HH12]	ASN^228^ [OD1]
ARG^1495^ [HH22]	ASN^228^ [OD1]
ARG^1496^ [HH12]	ARG^106^ [O]
ARG^1496^ [HH11]	LEU^104^ [O]
ARG^1496^ [HH22]	ARG^106^ [O]
ARG^1497^ [O]	ASN^146^ [HD21]
ARG^1497^ [H]	ASN^146^ [OD1]
ARG^1497^ [HH11]	GLN^181^ [OE1]
**Hydrogen bonds (minor site)**	
ARG^1495^ [H]	ASN^361^ [OD1]
ARG^1495^ [HH22]	GLU^396^ [OE1]
ARG^1496^ [HH22]	ASN^283^ [OD1]
ARG^1497^ [HH12]	GLU^354^ [OE2]
ARG^1497^ [HH21]	ASN^319^ [OD1]
ARG^1495^ [O]	ASN^361^ [HD21]
**Salt bridges (minor site)**	
LYS^1494^ [NZ]	ASP^325^ [OD1]
ARG^1495^ [NH1]	GLU^396^ [OE1]
ARG^1495^ [NH2]	GLU^396^ [OE1]
ARG^1495^ [NH2]	GLU^396^ [OE2]
ARG^1497^ [NH1]	GLU^354^ [OE1]
ARG^1497^ [NH1]	GLU^354^ [OE2]
ARG^1497^ [NH2]	GLU^354^ [OE1]
**Salt bridges (major site)**	
GLU^1491^ [OE1]	ARG^238^ [NH1]
LYS^1494^ [NZ]	ASP^192^ [OD1]

## Data Availability

The structure has been deposited in the Protein Data Bank (PDB) under the accession code 9PYR.

## Supplementary Materials

The following supporting information can be downloaded at: https://www.mdpi.com/article/10.3390/v17091279/s1, Figure S1: Alignment of human and abalone IMPα amino acid sequences. (a) Human IMPα1 and abalone IMPα1 like (58.72% homology); (b) mouse IMPα1 (named as IMPα2) and abalone IMPα1 like (57.79% homology); (c) human IMPα3 and abalone IMPα4 like (75.62% homology); (d) human IMPα5 and abalone IMPα6-like (73.03% homology); (e) human IMPα7- and abalone IMPα6-like (69.72% homology). Alignment was performed using the MAFFT L-INS-I algorithm within Geneious Prime (version 7.388). Conserved major (P2) and minor (P2’) binding sites of human and abalone IMPα are highlighted in the red box within the alignment; Figure S2: Conservation of the major-NLS binding between mouse IMP⍺2 and abalone IMP⍺1. Residues 68-520 of abalone (*Haliotis asinina*) IMP⍺1 (GenBank accession no. XP_067674051.1) were modelled using AlphaFold 3. (a) Superimposed structures of mouse IMP⍺2 (grey cartoon) and *H. asinina* IMP⍺1 (magenta cartoon) with the HaHV1 NLS shown as sticks. (b) Close-up of specific interactions identified in mouse IMP⍺2 crystal structure, with interacting IMP residues shown as sticks, highlighting strict conservation at the NLS-binding site.
