# Supplementary material for: Structural Insights into the Nuclear Import of Haliotid Herpesvirus 1 Large Tegument Protein Homologue
Source: Viruses. 2025 Sep 20;17(9):1279. doi: 10.3390/v17091279 (PMC12474298; doi:10.3390/v17091279)
Supplement: Supplementary file 1 [file viruses-17-01279-s001.zip › viruses-3871104-supplementary.pdf]

Structural insights into the nuclear import of haliotid herpesvirus 1 large tegument protein homolog

Babu Kanti Nath[1]*, Crystall M. D. Swarbrick[1], Renate H. M. Schwab[1], Daryl Ariawan[3], Ole Tietz[3], Jade K. Forwood[1,2]* and Subir Sarker[4,5]*

[1] Biosecurity Research Program and Training Centre, Gulbali Institute, Charles Sturt University, Wagga Wagga, NSW, Australia. Email: B.K.N., bnath@csu.edu.au; C.M.D.S., cswarbrick@csu.edu.au; R.H.M.S., rschwab@csu.edu.au; J.K.F., jforwood@csu.edu.au

[2] Training Hub Promoting Regional Industry and Innovation in Virology and Epidemiology, Gulbali Institute, Charles Sturt University, Wagga Wagga, New South Wales 2678, Australia. Email; J.K.F., jforwood@csu.edu.au

[3] Dementia Research Centre, Macquarie Medical School, Faculty of Medicine, Health and Human Sciences, Macquarie University, North Ryde, Sydney, NSW 2109, Australia. Email: R.B., reuben.blades@hdr.mq.edu.au; D.A., daryl.ariawan@mq.edu.au; O.T., ole.tietz@mq.edu.au

[4] Biomedical Sciences & Molecular Biology, College of Medicine and Dentistry, James Cook University, Townsville, QLD 4811, Australia. Email: S.S., subir.sarker@jcu.edu.au

[5] Department of Microbiology, Anatomy, Physiology and Pharmacology, School of Agriculture, Biomedicine and Environment, La Trobe University, Melbourne, Victoria 3086, Australia.

* Correspondence: bnath@csu.edu.au; jforwood@csu.edu.au; subir.sarker@jcu.edu.au

(a)

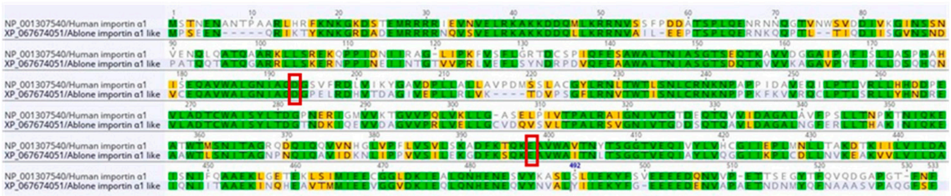

(b)

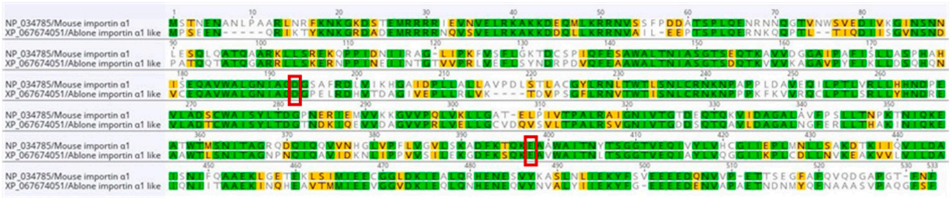

(c)

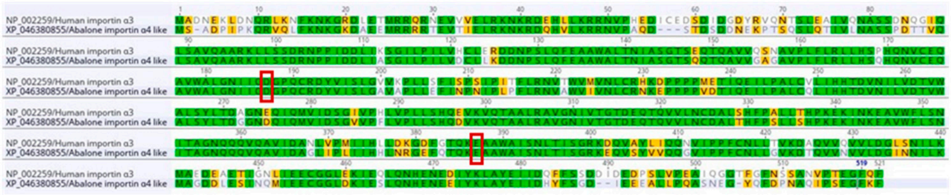

(d)

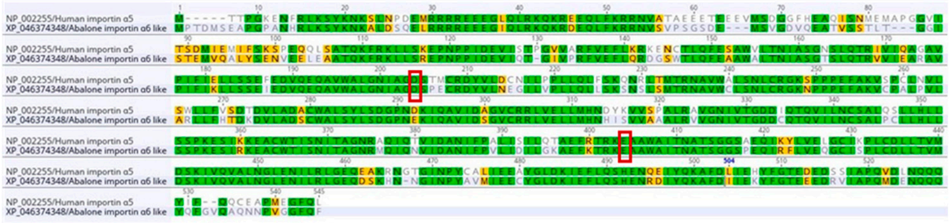

(e)

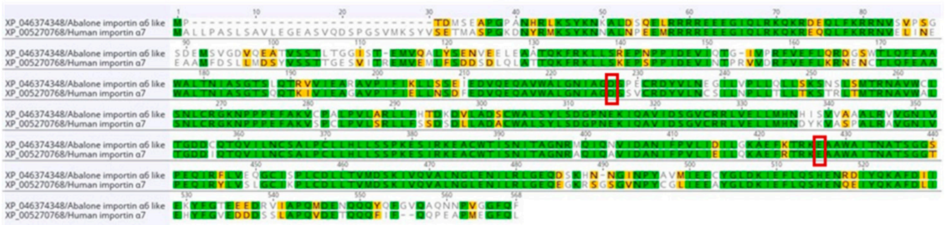

Supplementary Figure S1: Alignment of human and abalone IMPα amino acid sequences. (a) Human IMPα1 and abalone IMPα1 like (58.72% homology) (b) Mouse IMPα1 (named as IMPα2) and abalone IMPα1 like (57.79% homology) (c) Human IMPα3 and abalone IMPα4 like (75.62% homology) (d) Human IMPα5 and abalone IMPα6 like (73.03% homology) (e) Human IMPα7 and abalone IMPα6 like (69.72% homology). Alignment was performed using the MAFFT L-INS-I algorithm within Geneious Prime (version 7.388). Conserved major (P2) and minor (P2´) binding sites of human and abalone IMPα are highlighted in the red box within the alignment.

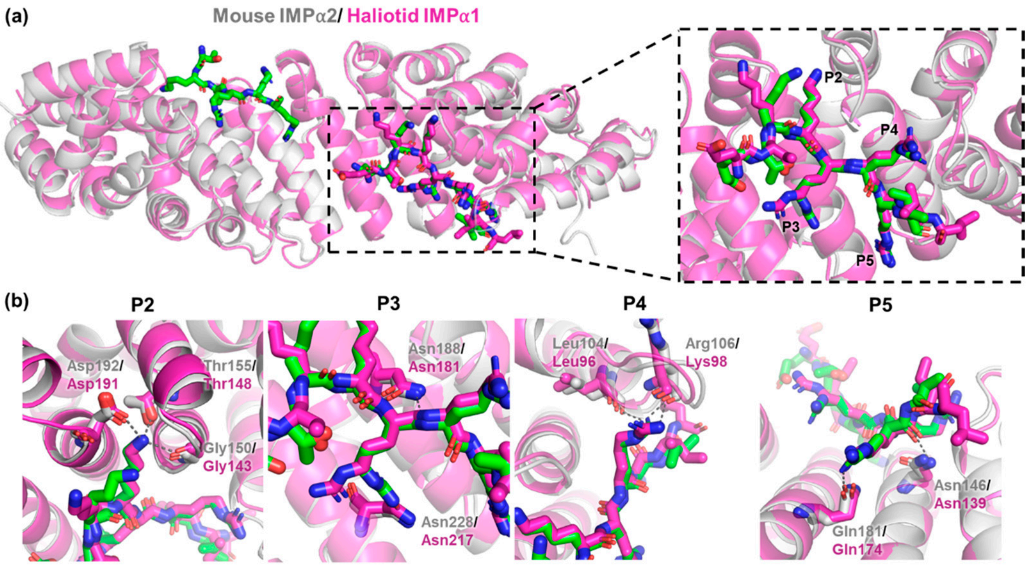

Supplementary Figure S2: Conservation of the major-NLS binding between mouse IMPα2 and abalone IMPα1. Residues 68-520 of abalone (*Haliotis asinina*) IMPα1 (GenBank accession no. XP_067674051.1) were modelled using AlphaFold 3. **(a)** Superimposed structures of mouse IMPα2 (grey cartoon) and *H. asinina* IMPα1 (magenta cartoon) with the HaHV1 NLS shown as sticks. **(b)** Close-up of specific interactions identified in mouse IMPα2 crystal structure, with interacting IMP residues shown as sticks, highlighting strict conservation at the NLS-binding site.
